# Graves’ disease, its treatments, and the risk of atrial fibrillation: A Korean population-based study

**DOI:** 10.3389/fendo.2022.1032764

**Published:** 2022-11-01

**Authors:** Yoon Young Cho, Bongseong Kim, Dughyun Choi, Chul-Hee Kim, Dong Wook Shin, Jee Soo Kim, Seung-Jung Park, Sun Wook Kim, Jae Hoon Chung, Kyungdo Han, Tae Hyuk Kim

**Affiliations:** ^1^ Division of Endocrinology and Metabolism, Department of Medicine, Soonchunhyang University Bucheon Hospital, Bucheon, South Korea; ^2^ Department of Statistics and Actuarial Science, Soongsil University, Seoul, South Korea; ^3^ Department of Family Medicine, Samsung Medical Center, Sungkyunkwan University, Seoul, South Korea; ^4^ Department of Clinical Research Design and Evaluation, Samsung Advanced Institute for Health Science and Technology, Sungkyunkwan University, Seoul, South Korea; ^5^ Division of Endocrine Surgery, Department of Surgery, Samsung Medical Center, Sungkyunkwan University, Seoul, South Korea; ^6^ Division of Cardiology, Department of Medicine, Heart Vascular Stroke Institute, Samsung Medical Center, Sungkyunkwan University, Seoul, South Korea; ^7^ Division of Endocrinology and Metabolism, Department of Medicine, Samsung Medical Center, Sungkyunkwan University, Seoul, South Korea

**Keywords:** graves’ disease, atrial fibrillation, epidemiology, anti-thyroid drug (ATD), radioactive iodine therapy, surgery

## Abstract

**Background:**

Atrial fibrillation (AF) is occasionally diagnosed in individuals with Graves’ disease. Definite treatments, including radioactive iodine therapy (RAIT) or surgery might lower the risk of AF in the literature. However, no studies have compared the effects of anti-thyroid drugs (ATDs), RAIT, and surgery on the risk of AF.

**Methods:**

This retrospective cohort study included 94,060 newly diagnosed Graves’ disease patients and 470,300 controls from the Korean National Health Insurance database. The incidence of AF was evaluated in patients and controls. Patients were categorized based on treatment method into ATD (95.6%), RAIT (3.5%), and surgery (0.9%) groups. In the ATD group, the dose and duration of ATDs were calculated for each patient. In the RAIT and surgery groups, remission was defined as levothyroxine prescription.

**Results:**

Graves’ disease patients had a 2.2-fold higher risk of developing AF than controls. Regardless of demographic factors, the patient group had a consistently higher risk of AF than controls, with the highest risk of AF (HR, 5.49) in the younger patient group. The surgery group had a similar risk of AF compared with controls, whereas the ATD (HR, 2.23) and RAIT (HR, 2.00) groups had increased risks of AF, even in patients reaching hypothyroid status after RAIT. Patients with higher dose or longer treatment duration of ATDs were at greater risk of AF.

**Conclusion:**

We observed differing risks of AF according to methods of treatment for Graves’ disease, and that definite treatment can be an option for subjects needing sustained medical treatment considering the risk of AF.

## Introduction

Graves’ disease is the most common cause of hyperthyroidism with a prevalence of 0.3–0.5% in iodine-sufficient areas (1, 2). Effects of excessive thyroid hormones on the musculature and conduction system of the heart, vascular musculature, and coagulation are associated with increased risk of cardiovascular manifestations (3). Franklyn et al. demonstrated that in 15,968 person-years (PYs) of follow-up, cardiovascular manifestations, including atrial fibrillation (AF), myocardial infarction (MI), ischemic stroke, and heart failure (HF), can contribute to an up to 20% higher risk of mortality in individuals with persistent thyrotoxicosis after radioactive iodine therapy (RAIT) compared to the general population (4). Okosieme et al. reported that patients with Graves’ disease had a higher all-cause mortality (hazard ratio [HR], 1.23, 95% CI: 1.06–1.42) and major adverse cardiovascular event (HR, 2.47, 95% CI: 2.16–2.81) in 25,266 PYs compared to controls in 103,374 PYs (5). Cardiovascular sequelae of Graves’ disease constitute a significant socioeconomic burden, individually and socially (6).

AF is the most common type of sustained cardiac arrhythmia, and its prevalence has increased over recent decades (7, 8). The prevalence of AF is approximately 2% in Western countries (8, 9) but is lower at 1% in Asian countries (10–12). With aging of the population, the incidence of AF is expected to increase by 1.5–2 fold during the next few decades (13, 14). Clinical implication of AF is that it is a well-known independent risk factor for ischemic stroke (15). AF is associated with a 5-fold risk of ischemic stroke, accounting for approximately 20% of all stroke cases (15).

The risk of AF might be lowered by appropriate treatment in individuals with Graves’ disease. Epidemiologic studies have shown that hypothyroid status by definite treatment, including RAIT or surgery, can lower the risk of AF in patients with Graves’ disease (4, 16–18). However, insufficient treatment of Graves’ disease is related to a higher risk of AF compared to that of controls, regardless of treatment method (4, 16–18). Boelaert et al. reported a 1.3-fold higher mortality in medically treated patients for hyperthyroidism compared to controls, whereas hypothyroid patients after RAIT had a similar mortality rate (standardized mortality ratio [SMR], 0.98) in 12,868 PYs of follow-up (18). In their study, increased all-cause mortality mainly reflected increased circulatory death and AF was associated with mortality (18). Ryödi et al. observed an increased risk of AF in patients who had not resulting in hypothyroidism after RAIT (HR, 1.44, 95% CI: 1.18–1.75) compared to surgically treated patients; in contrast, the risks of AF were comparable in patients who had received thyroidectomy and RAIT leading to hypothyroidism (17). Previous studies examining the risk of AF according to treatment method and clinical course of Graves’ disease are limited, and no studies have compared the risks of AF among patients who had received differing treatments of anti-thyroid drug (ATD), RAIT, or surgery. In addition, previous studies were conducted in Western countries (4, 16, 17), where the prevalence of AF is generally higher than it is in Asian countries (10–12). Therefore, we evaluated the risk of AF in the Korean population with Grave’ disease according to treatment methods and clinical course using nationally representative data from the National Health Information Database (NHID).

## Methods

### Data source

Data were retrieved from the Korean NHID, which is maintained by the National Health Insurance Service (NHIS), the only public medical insurance system in Korea and covers all Korean citizens. The NHID contains nationally representative data and includes diagnostic codes based on the standard International Classification of Diseases (ICD)-10; prescriptions; patient demographic data, including age, sex, and region; and data on billing for medical expenses (19). The NHID is accessible online at designated analysis centers based on fulfillment of strict criteria (https://nhiss.nhis.or.kr/). The retrospective study protocol was approved by the Institutional Review Board of Samsung Medical Center (2019-01-034). The requirements for patient approval and informed consent were waived because we used publicly available and deidentified patient data. All procedures for this study complied with relevant patient confidentiality guidelines.

### Study population

We identified 103,932 individuals newly diagnosed with Graves’ disease between January 2010 and December 2014 using the ICD-10 code for hyperthyroidism (E05). To find cases with Graves’ disease excluding transient thyrotoxicosis, individuals who had received ATD ≥60 consecutive days or who had undergone thyroid surgery or RAIT were enrolled. The prescription codes (Anatomical Therapeutic Chemical [ATC] code) for methimazole (H03BB02), carbimazole (H03BB01), and propylthiouracil (H03BA02) were used. This method of case detection has been used in previous epidemiologic studies of Graves’ disease in Korea (2, 20). To find newly diagnosed cases of Graves’ disease, a washout period was applied from January 2006 to the time of study enrollment. Patients younger than 20 years, diagnosed with thyroid carcinoma (C73.9), or with missing data were excluded. We also excluded with individuals with previous AF based on ICD-10 codes (I480–I484, and I489) during the washout period. Finally, we included 94,060 Graves’ disease patients. For a control population, we identified 470,300 individuals from NHID who were matched to patients on age and sex, at a ratio of five controls per patient, and controls were excluded for the diagnostic codes of any cause of hyperthyroidism. The study period ended in December 2018.

### Treatment modality for Graves’ disease and prescription of ATDs

The methods of treatment for Graves’ disease were classified according to whether patients had received ATDs for ≥60 consecutive days (medical treatment), had undergone RAIT, or had undergone thyroid surgery. Patients who had undergone RAIT or surgery for Graves’ disease were assigned to the RAIT group or surgery group, respectively, regardless of whether they had received ATDs.

The cumulative doses of ATDs and duration of ATD treatment were calculated for each patient using ATC codes for drug prescriptions. The cumulative ATD dose was classified as <5,743 mg (lowest tertile), 5,743–22,055 mg (middle tertile), and >22,055 mg (highest tertile). The ATD treatment duration was classified as <11.5 months (lowest tertile), 11.5–33.4 months (middle tertile), and >33.4 months (highest tertile).

In the ATD group, remission was defined when patients had discontinued the ATD prescription and no more prescriptions for ATDs were started during the study period. In the RAIT and surgery groups, remission was defined as hypothyroid patients taking levothyroxine after RAIT or surgery.

### Definition of AF

AF was diagnosed based on ICD-10 codes (I480–I484, and I489). To ensure diagnostic accuracy and exclude transient AF, we included patients with AF only when it was a discharge diagnosis or confirmed more than twice in an outpatient clinic (12, 21). To identify non-valvular AF, subjects with mitral stenosis (I50, I52, and I59) or preexisting mechanical heart valves (Z952–Z945) were excluded.

### Covariates

Comorbidities, including diabetes, hypertension, and dyslipidemia, were defined based on ICD-10 codes for diagnoses and one or more drug prescriptions for related diseases. Household income was classified into quartiles (Q1–Q4) and absolute poverty, defined as monthly household income less than the minimum cost of living.

### Statistics

Descriptive statistics (mean, standard deviation [SD], number, and percentage) were tabulated for baseline characteristics. Categorical variables were compared using the chi-square test. The independent *t*-test was used for continuous variables. To compare baseline characteristics among three treatment groups, chi-square test was used for categorical variables and one-way ANOVA for continuous variables. Ordinal variable, such as household income was compared using the chi-square test. Conventional Cox proportional hazard regression analyses were performed to evaluate the relationship between Graves’ disease and incident AF. The index date for Cox proportional hazard regression analyses as below: the first prescription date of ATDs in the ATD group and the date of the first RAIT or surgery in the RAIT or surgery groups. The HR was not adjusted in model 1, while model 2 was adjusted for household income, diabetes, hypertension, and dyslipidemia. P-values <0.05 were considered significant. All statistical analyses were performed using SAS software (version 9.4; SAS Institute Inc., Cary, NC, USA).

## Results

### Baseline characteristics

This study included 94,060 Graves’ disease patients and 470,300 matched controls. The mean age was 46 years, the majority of the sample (90%) was <65 years, and 28% of subjects in both groups were men. Graves’ disease patients had higher prevalence of diabetes, hypertension, and dyslipidemia (**Table 1**). The mean follow-up period was 7.0 years in both groups.

**Table 1:** Baseline characteristic of patients with Graves’ disease and controls.

Variables	Controls (n=470,300)	Graves’ disease (n=94,060)	P-value	ATD (n=89,991)	RAIT (n=3,261)	Surgery (n=808)	P-value*
Male sex	131,905 (28%)	26,381 (28%)	1	25,117 (28%)	1,083 (33%)	181 (22%)	< 0.01
Mean age, years	46 ± 14	46 ± 14	1	46 ± 14	44 ± 13	44 ± 14	< 0.01
Age ≥65 years	48,375 (10%)	9,675 (10%)	1	9,348 (10%)	245 (8%)	82 (10%)	< 0.01
Household income			< 0.01				< 0.01
Absolute poverty	25,166 (5%)	5,423 (6%)		5,185 (6%)	182 (6%)	56 (7%)	
First quartile	88,997 (19%)	17,033 (18%)		16,287 (18%)	594 (18%)	152 (19%)	
Second quartile	98,134 (21%)	18,737 (20%)		17,891 (20%)	664 (20%)	182 (23%)	
Third quartile	117,161 (25%)	23,388 (25%)		22,402 (25%)	774 (24%)	212 (26%)	
Fourth quartile	140,842 (30%)	29,479 (31%)		28,226 (31%)	1,047 (32%)	206 (25%)	
Comorbidity							
Diabetes	24,749 (5%)	8,141 (9%)	< 0.01	7,827 (9%)	229 (7%)	85 (11%)	< 0.01
Hypertension	73,064 (16%)	25,605 (27%)	< 0.01	24,144 (27%)	1,165 (36%)	296 (37%)	< 0.01
Dyslipidemia	42,288 (9%)	10,044 (11%)	< 0.01	9.719 (11%)	247 (8%)	78 (10%)	< 0.01

ATD, anti-thyroid drug; RAIT, radioactive iodine therapy.

*P-value was calculated among three groups according to the treatment modality.

The vast majority of patients (95.6%) had been treated with ATDs, whereas patients who had undergone RAIT or surgery accounted for only 4% of the Korean population (3.5% for RAIT and 0.9% for surgery). The mean age of the ATD group was higher than the other two groups (46 vs. 44 years); however, the percentages of population ≥65 years were similar in the ATD and surgery groups (10% in each group). The surgery group had a higher percentage of female and higher prevalences of diabetes and dyslipidemia, compared to those of the ATD and RAIT groups (**Table 1**).

### Association between Graves’ disease and incident AF

**Figure 1:**
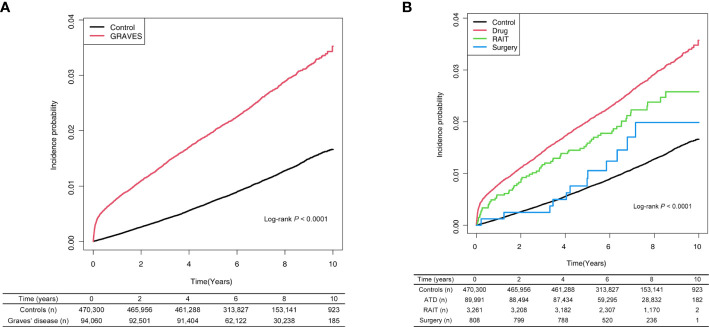
**(A)** Risk of atrial fibrillation among patients with Graves’ disease and controls. **(B)** Risk of atrial fibrillation among patients with Graves’ disease according to treatment modality. ATD, anti-thyroid drug; RAIT, radioactive iodine therapy.

Among the 94,060 Graves’ disease patients, 2,444 cases of incident AF were diagnosed during 654,186 PYs of follow-up. Relative to controls, Graves’ disease patients showed a greater than two-fold risk of incident AF (HR, 2.37, 95% CI: 2.26–2.48) (**Table 2**). Graves’ disease patients had increased risk of developing AF after adjusting for age, sex, household income, and comorbidities (HR, 2.20, 95% CI: 2.09–2.31) (**Table 2**; **Figure 1A**).

**Table 2:** Risk of atrial fibrillation among patients with Graves’ disease according to treatment modality.

Treatment modality	n	AF	PYs	IR per 1,000 PYs	Model 1	Model 2
					Hazard ratio (95% CI)	Hazard ratio (95% CI)
Controls	470,300	5,209	3,299,357	1.58	Reference	Reference
Graves’ disease	94,060	2,444	654,186	3.74	2.37 (2.26–2.48)	2.20 (2.09–2.31)
ATD	89,991	2,363	625,633	3.78	2.39 (2.28–2.51)	2.23 (2.11–2.32)
RAIT	3,261	69	23,057	2.99	1.89 (1.49–2.40)	2.00 (1.58–2.54)
Surgery	808	12	5,496	2.18	1.39 (0.79–2.44)	1.43 (0.81–2.52)

AF, atrial fibrillation; PYs, person-years; IR, incidence rate; ATD, anti-thyroid drug; RAIT, radioactive iodine therapy.

Model 1: not adjusted.

Model 2: adjusted for household income and comorbidities.

Subgroup analyses were performed according to age, sex, and comorbidities (**Table 3**). Absolute incidence of AF was higher in those aged ≥65 years; males; and individuals with diabetes, hypertension, or dyslipidemia. After adjusting for demographic variables, patients with Graves’ disease had significantly higher risk of AF compared to controls. In particular, younger patients (20–39 years) and patients without diabetes, hypertension, or dyslipidemia had higher risk for developing AF compared to Graves’ disease patients who were elderly or had other comorbidities (P for interaction <0.01).

**Table 3:** Subgroup analyses of atrial fibrillation risk among patients with Graves’ disease and controls.

Subgroup	Graves’ disease	n	AF	PYs	IR per 1,000 PYs	Hazard ratio (95% CI)	P for interaction
20–39 years	No	162,090	337	1,168,751	0.29	Reference	< 0.01
	Yes	32,418	413	232,145	1.78	5.49 (4.72–6.38)	
40–64 years	No	259,835	2,566	1,821,828	1.41	Reference	
	Yes	51,967	1,272	361,434	3.52	2.32 (2.17–2.49)	
≥65 years	No	48,375	2,306	308,778	7.47	Reference	
	Yes	9,675	759	60,607	12.52	1.58 (1.45–1.71)	
Male	No	131,905	1,926	916,703	2.10	Reference	0.17
	Yes	26,381	947	181,289	5.22	2.31 (2.14–2.50)	
Female	No	338,395	3,283	2,382,654	1.38	Reference	
	Yes	67,679	1,497	472,897	3.17	2.14 (2.01–2.28)	
No diabetes	No	445,551	4,429	3,137,363	1.41	Reference	< 0.01
	Yes	85,919	2,036	600,705	3.39	2.30 (2.19–2.43)	
Diabetes	No	24,749	780	161,994	4.81	Reference	
	Yes	8,141	408	53,481	7.63	1.73 (1.54–1.95)	
No hypertension	No	397,236	2,746	2,811,075	0.98	Reference	< 0.01
	Yes	68,455	1,217	480,464	2.53	2.71 (2.53–2.90)	
Hypertension	No	73,064	2,463	488,283	5.04	Reference	
	Yes	25,605	1,227	173,722	7.06	1.77 (1.66–1.90)	
No dyslipidemia	No	428,012	4,063	3,021,026	1.34	Reference	< 0.01
	Yes	84,016	1,988	588,736	3.38	2.67 (2.24–2.50)	
Dyslipidemia	No	42,288	1,146	278,332	4.12	Reference	
	Yes	10,044	456	65,450	6.97	1.67 (1.50–1.86)	

AF, atrial fibrillation; PYs, person-years; IR, incidence rate.

Hazard ratio was adjusted for household income and comorbidities.

### Associations between Graves’ disease and incident AF according to treatment modality

Relative to controls, the ATD group had a 2.2-fold higher risk of developing AF (HR: 2.23, 95% CI: 2.11–2.32), and the RAIT group had a 2-fold higher risk of AF (HR: 2.00, 95% CI: 1.58–2.54). However, the risk of AF was not significant in the surgery group (HR: 1.43, 95% CI: 0.81–2.52) (**Table 2**; **Figure 1B**).

In the ATD group, patients who did not achieve remission had a 2.2-fold higher risk of developing AF (HR: 2.24, 95% CI: 2.13–2.36), with 2,306 AF in 86,349 patients, whereas the patients who did achieve remission had a 1.4-fold higher risk of AF (HR: 1.44, 95% CI: 1.11–1.87), with 57 AF in 3,642 patients. In the RAIT group, remitted patients had a 2.0-fold higher risk of incident AF (HR: 2.04, 95% CI: 1.51–2.75), with 43 AF in 2,033 patients; patients who did not experience remission had a 1.9-fold higher risk of AF with 26 AF in 1,228 patients (HR: 1.95, 95% CI: 1.33–2.86, data not shown). In the surgery group, all patients achieved remission that is, they were supplemented with levothyroxine due to hypothyroid status after surgery.

### Associations between Graves’ disease and AF according to cumulative ATD dose and treatment duration

**Figure 2:**
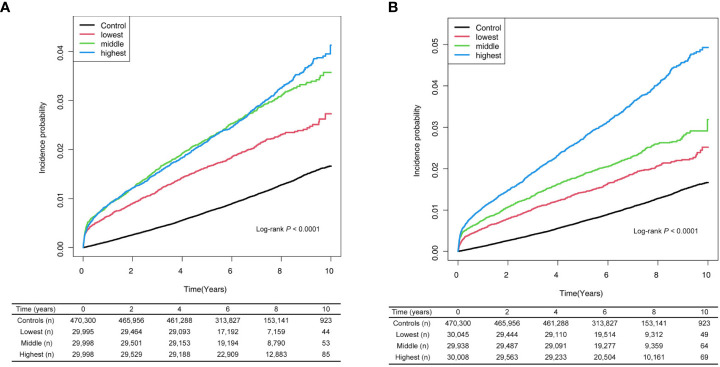
Risk of atrial fibrillation among patients with Graves’ disease according to cumulative dose **(A)** and treatment duration **(B)** of anti-thyroid drug. Cumulative doses were <5,743 mg (lowest), 5,743–22,055 mg (middle), and >22,055 mg (highest). Treatment durations were <11.5 months (lowest tertile), 11.5–33.4 months (middle tertile), and >33.4 months (highest tertile).

When we classified Graves’ disease patients according to cumulative ATD dose and treatment duration, an increased risk of AF was observed across the Graves’ disease subgroups relative to the controls. In particular, a higher cumulative dose or longer treatment duration with ATDs were associated with the increased risks of developing AF (**Table 4**; **Figure 2**).

**Table 4:** Risk of atrial fibrillation among patients with Graves’ disease according to cumulative dose and treatment duration of anti-thyroid drug.

	n	AF	PYs	IR per 1,000 PYs	Model 1	Model 2	P for interaction
					Hazard ratio (95% CI)	Hazard ratio (95% CI)	
Controls	470,300	5,209	3,299,357	1.58	Reference	Reference	
Cumulative dose							< 0.01
Lowest	29,995	595	196,805	3.02	1.93 (1.77–2.09)	1.60 (1.47–1.74)	
Middle	29,998	835	205,483	4.06	2.58 (2.39–2.77)	2.42 (2.25–2.61)	
Highest	29,998	933	223,345	4.18	2.63 (2.45–2.82)	2.66 (2.48–2.86)	
Treatment duration							< 0.01
Lowest	30,045	552	207,042	2.67	1.69 (1.55–1.84)	1.62 (1.48–1.77)
Middle	29,938	693	206,628	3.35	2.13 (1.96–2.30)	2.10 (1.94–2.27)
Highest	30,008	1,118	211,963	5.27	3.34 (3.13–3.56)	2.83 (2.65–3.02)

AF, atrial fibrillation; PYs, person-years; IR, incidence rate.

Cumulative doses were <5,743 mg (lowest), 5,743–22,055 mg (middle), and >22,055 mg (highest).

Treatment durations were <11.5 months (lowest tertile), 11.5–33.4 months (middle tertile), and >33.4 months (highest tertile).

Model 1: not adjusted.

Model 2: adjusted for household income and comorbidities

## Discussion

AF is the common cardiovascular manifestation of Graves’ disease and the risk can be lowered by appropriate treatment of Graves’ disease. In our sample, individuals with Graves’ disease had a 2.2-fold higher risk of incident AF (3.74 IR per 1,000 PYs) compared to controls (1.58 IR per 1,000 PYs). Elevated risk of AF was observed in all subgroups (HRs, ranging from 1.58 to 5.49), and HR was most prominent in the younger population (20–39 years). Among the three treatment methods, the surgical group was not at a significant risk of AF compared to controls, although the surgically treated patients accounted less than 1% of whole population. The risk of AF was elevated after RAIT even in hypothyroid patients. In the ATD group, the risk was more marked in those with higher cumulative dose or longer treatment duration. Based on our results, hyperthyroidism needing sustained medical treatment was associated with the greater risk of incident AF and the AF risk was not significantly elevated in surgically treated patients.

Evidence supporting the importance of definitive treatment of Graves’ disease on cardiovascular outcomes has increased. Boelaert et al. compared mortality of patients with hyperthyroidism receiving medical therapy and those undergoing RAIT among samples from England and Wales (*n* = 2,668) (18). They observed increased all-cause mortality in medically treated patients (SMR, 1.30) or patients with RAIT not achieving hypothyroidism (SMR, 1.24), whereas patients with RAIT experiencing hypothyroidism had risk comparable with that of the general population (SMR, 0.98). Increased mortality was largely caused by circulatory death, and AF at presentation was related to higher mortality (*P* = 0.02) (18). Ryödi et al. compared the cardiovascular diseases (CVDs) of patients with hyperthyroidism receiving RAIT or surgery in the Finnish population (*n* = 6,148) and found that the risk of CVDs was not elevated in the surgical group (17). However, the RAIT-treated group, especially patients who did not experience hypothyroidism after treatment had a higher risk of CVDs, including AF. Okosieme et al. examined data from 4,189 United Kingdom patients with Graves’ disease and concluded that the RAIT group with resolved hyperthyroidism had lower mortality (HR, 0.50) compared to the ATD group, but the RAIT group with unresolved hyperthyroidism did not (HR, 1.55) (5). Using a database of a Danish population (*n* = 275,467), Lillevang-Johansen et al. reported that increased CVD risk was observed in untreated (odds ratio [OR], 1.25) or insufficiently treated patients with decreased TSH (OR, 1.09), although they did not analyze data according to treatment method (16). However, previous studies did not deal with CVD outcomes of Graves’ disease based on comparison of three treatment methods.

In line with previous epidemiologic studies (5, 16–18), we observed similar risk of AF in the surgical group and controls. However, RAIT did not eliminate additional risk of AF, even in patients achieving hypothyroidism. Surgery is most effective (>99%) for Graves’ disease remission, followed by RAIT (>80%) (22, 23). Levothyroxine replacement should be started immediately after thyroidectomy, whereas achievement of remission takes several months following RAIT (23). Differences in remission rate and time to remission seem to result in different risks of incident AF in the surgery and RAIT groups. In Korea, as an iodine replete area, RAIT is preferred as the second most common therapy for Graves’ disease after ATDs. Recently, Part et al. reported a 63% of the remission rate of Graves’ disease after one year of the first RAIT, which was lower than that of success rate (80–90%) in the United States (24); difference in success rate of RAIT among regions suggest that the effects of RAIT on the risk of incident AF also differ among countries. Patients needing sustained medical treatment with higher cumulative dose and duration were at higher risk for AF. Medical treatment is most commonly used as initial therapy for Graves’ disease (20, 22, 23) but has the lowest efficacy, with a 30–40% remission rate and gradually increasing treatment failure rate during follow-up (22, 23). Recently, Song et al. reported that longer ATD treatment duration (>5 years) was associated with higher risk of HF (HR, 1.51) (25), similar to our data. Their study was the only one evaluating the relationship between duration of ATD use and CVD outcomes prior to our study. However, Song et al. included 16,882 patients who received ATD >24 months using the Korean NHID database (25), and their sample was smaller than that in the present study (*n* = 94,060). Finally, they did not include patients who underwent surgery. Recently, Elnahla et al. demonstrated significant and rapid improvements of hypertension (45% in surgical treatment vs. 18% in medical treatment), tachyarrhythmia (86% vs. 66%), and HF (75% vs. 50%) among patients receiving total thyroidectomy for Graves’ disease compared to those with ATD therapy in a retrospective study (26). However, surgical treatment tends to be less cost-effective compared to medical therapy and RAIT based on studies conducted in the United States and United Kingdom (27), and postsurgical complications, including hypoparathyroidism and recurrent laryngeal nerve injury can occur in 1–4% of patients (28, 29). Thus, considering pros and cons of surgical treatment for Graves’ disease, surgery can be an appropriate option for selected Graves’ disease patients.

We found that the younger population aged 20–39 years, who accounted for 34% of the entire Graves’ disease population, had an approximately 5-fold higher risk of incident AF compared to controls. Brito et al. demonstrated a higher risk of failed ATD therapy in younger Graves’ disease patients (<35 years) than in elderly patients (55–64 years) based on nationwide population-based data from the United States (22), with these observations probably related to higher disease activity or low compliance with long-standing medical treatment among younger patients. Thus, younger Graves’ disease patients who are expected to need longer durations and higher doses of ATD therapy might achieve better outcomes with more definite treatment than medical therapy for preventing CVD comorbidity and controlling Graves’ disease.

As expected, elderly patients, male subjects, or those with comorbidities had higher incidence of AF, even among controls. AF incidence tends to increase with age and presence of comorbidities, such as hypertension and diabetes, which are known risk factors for AF (12). Based on multivariate analysis, even among groups with several risk factors for AF, we found that Graves’ disease was an independent predisposing factor for AF (HR, 1.58–1.77).

The present study has several limitations. First, diagnosis of Graves’ disease is based on a combination of ICD-10 codes and prescriptions for medication and/or procedure codes of RAIT/thyroidectomy. We used consecutive prescriptions (≥60 days) of ATDs to exclude transient thyrotoxicosis, a definition adopted in previous epidemiologic studies in Korea (2, 20). Graves’ disease accounts for 82.7% of thyrotoxicosis, followed by subacute thyroiditis (13.3%) and painless thyroiditis (3.5%), whereas toxic adenoma is a rare cause (0.5%) in Korea (30). As we excluded transient thyrotoxicosis with consecutive use of ATDs thus, our results can plausibly be interpreted to reflect the effects of Graves’ disease. Second, the NHID does not provide information regarding thyroid function or autoantibody titers, limiting evaluation of exact status of thyroid function, disease activity, and remission status, and their effects on the risk of AF, which was one of major weak points in this study. Instead, we used prescription information for levothyroxine after RAIT or surgery to assess remission status, although these data alone are not sufficient for such a conclusion. Third, ATD was the major treatment method, and definite treatment groups accounted for only 4% of Graves’ disease patients, as expected. Seo et al. reported the treatment pattern for Graves’ disease in Korea, using 177,487 patients treated between 2007 and 2011; the majority of patients (91%) were treated medically only, followed by RAIT (8.1%) and surgery (0.9%) (20). Traditionally, countries in Eurasia, including Korea and Japan prefer medical treatment as initial therapy for Graves’ disease; in North America, RAIT had been preferred, although ATD (60%) has replaced RAIT (33%) as the most common therapy (22). With the large population-based sample used in this study, we included significant numbers of patients who received definite treatments (3,261 RAIT patients and 808 thyroidectomized patients) compared to previous epidemiologic studies (5, 17, 18, 25); however, deviation to the medically treated group and the small proportion of the surgically treated group weaken the statistical power in the surgery group. In addition, several clinical factors preferring surgical treatment, such as age and comorbidities may affect the risk of incident AF in spite of statistical correction for confounders. Therefore, to clarify the risk of AF in surgically treated subjects, further studies with larger population are warranted. Fourth, the importance of our results from an observational study, not from a randomized study might be attenuated and limited. However, due to low prevalence of Graves’ disease and patients’ or clinicians’ preference or indications for treatment modality, it might be difficult to plan randomized research for Graves’ disease. Fifth, we did not assess risks of other CVDs, including MI and stroke, or mortality because of difficulties in handling the various outcomes in our large cohort. Recently, evidence has shown that hyperthyroidism is related to atherosclerotic vascular disease (5, 16–18, 31), and further studies evaluating CVDs including MI, stroke, AF, and HF might provide more definite evidence that patients and clinicians can use to choose treatment methods for Graves’ disease. Although this study has such limitations, the strengths are the large population-based sample and the use of a sole cohort for comparing the effects of three treatment methods of Graves’ disease on risk of AF.

We found that patients with Graves’ disease had a 2-fold higher risk of incident AF compared to controls, and that younger patients are at a greater than 5-fold higher risk of AF. Based on the results of our study, the risks of AF were more prominent in higher dose and longer duration of medical treatment. Whereas, surgically treated individuals may be at similar risk of AF to controls, although the clinical meaning might be limited due to the small proportion of the surgically treated population. In aspect of the AF risk, effective treatment, such as definite treatment can be considered in Graves’ disease patients who are expected to need long-standing medical treatment.

## Data availability statement

The raw data supporting the conclusions of this article will be made available by the authors, without undue reservation.

## Ethics statement

Patient approval and informed consent for the use of publicly available and deidentified patient data were waived.

## Author contributions

TK, KH, and YC contributed to the design and conception of the study. KH and BK were responsible for acquisition of data. BK, TK, KH, and YC performed the statistical analyses and contributed to the interpretation of data. YC was the major contributor in writing the manuscript. All authors were involved in drafting the article and revising it critically, and all authors have read and approved the submitted version.

## Funding

This work was partly supported by the Soonchunhyang University Research Fund.

## Conflict of interest

The authors declare that the research was conducted in the absence of any commercial or financial relationships that could be construed as a potential conflict of interest.

## Publisher’s note

All claims expressed in this article are solely those of the authors and do not necessarily represent those of their affiliated organizations, or those of the publisher, the editors and the reviewers. Any product that may be evaluated in this article, or claim that may be made by its manufacturer, is not guaranteed or endorsed by the publisher.
